# Supplementary figures and images for: FAM111B Acts as an Oncogene in Bladder Cancer
Source: Cancers (Basel). 2023 Oct 24;15(21):5122. doi: 10.3390/cancers15215122 (PMC10648174; doi:10.3390/cancers15215122)

Figure S1

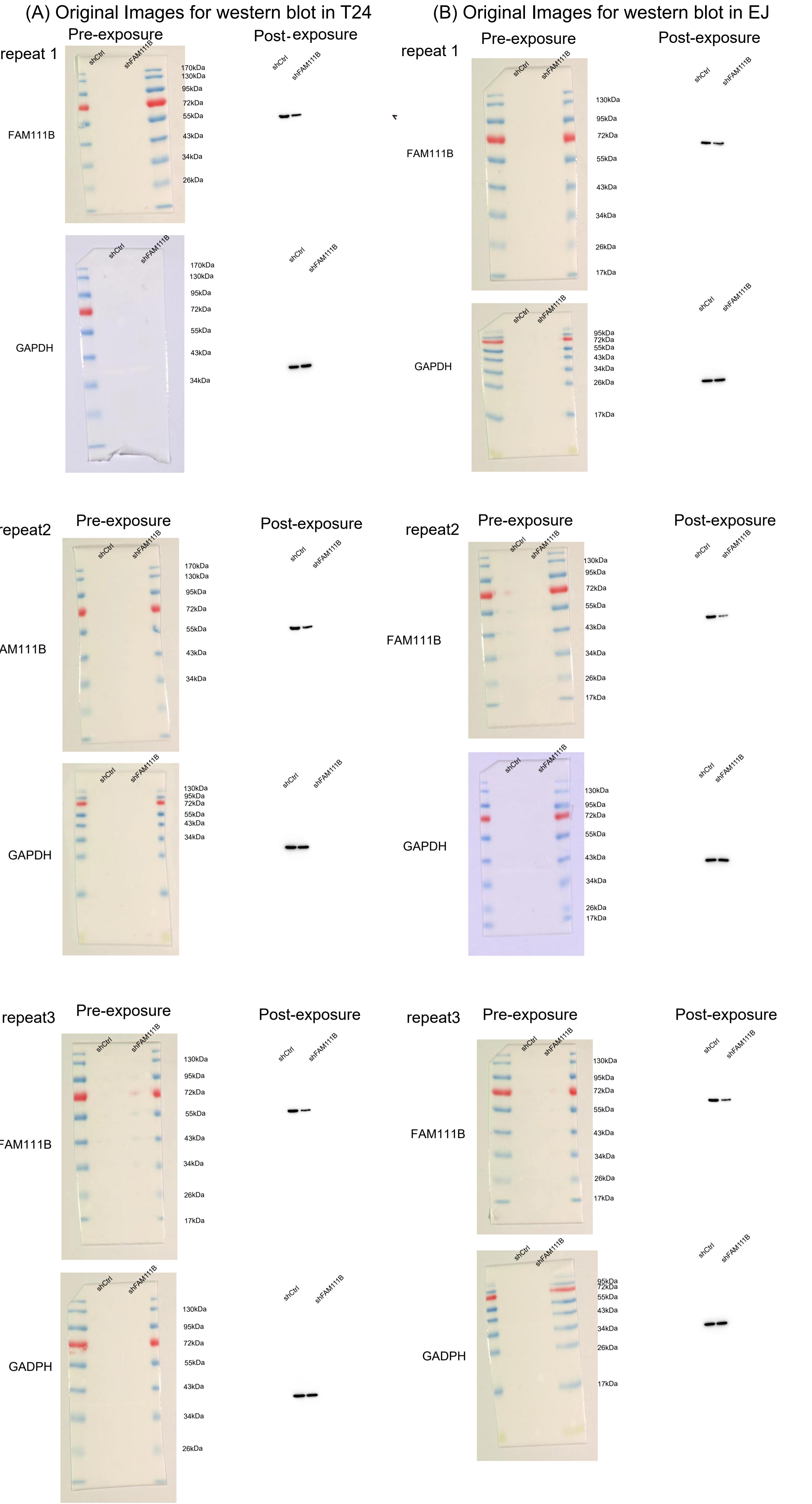

Supplement: Supplementary file 1 [file cancers-15-05122-s001.zip › Figure S1.pdf]

Figure S2

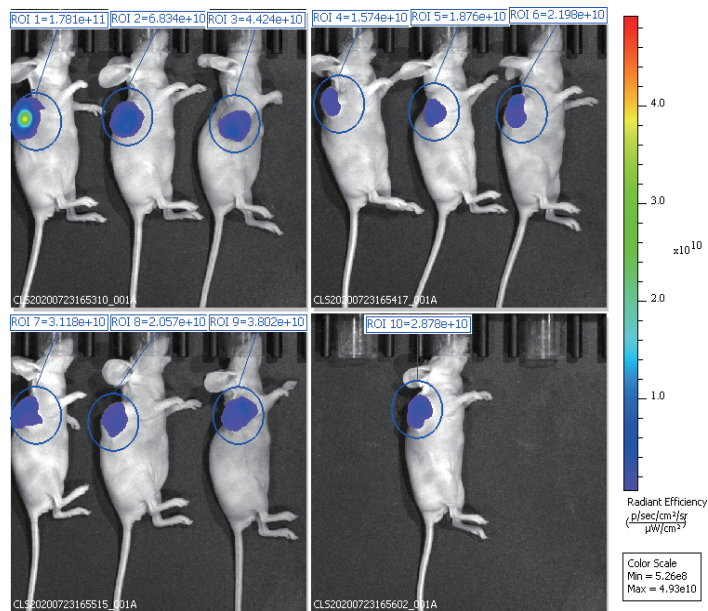

shCtrl

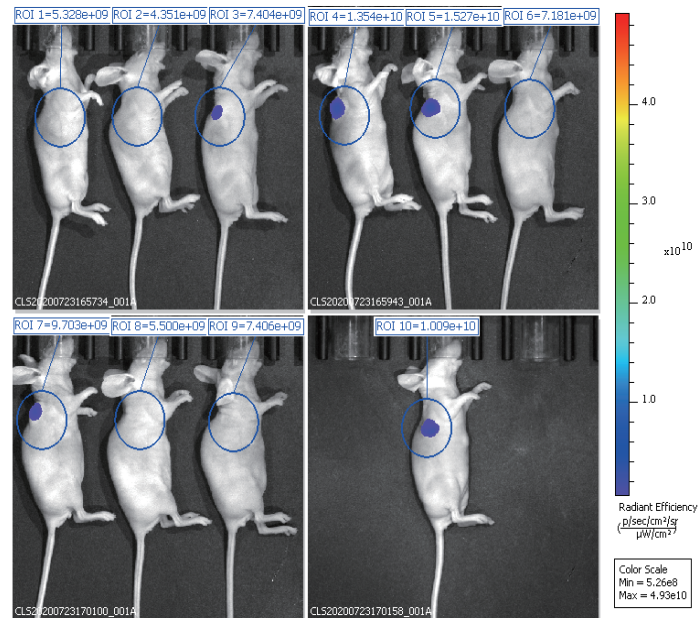

shFAM111B

Supplement: Supplementary file 1 [file cancers-15-05122-s001.zip › Figure S2.pdf]
